# Rheology-Assisted Microstructure Control for Printing Magnetic Composites—Material and Process Development

**DOI:** 10.3390/polym12092143

**Published:** 2020-09-20

**Authors:** Balakrishnan Nagarajan, Martin A.W. Schoen, Simon Trudel, Ahmed Jawad Qureshi, Pierre Mertiny

**Affiliations:** 1 Department of Mechanical Engineering, University of Alberta, 9211-116 St., NW Edmonton, AB T6G 1H9, Canada; bnagaraj@ualberta.ca (B.N.); ajquresh@ualberta.ca (A.J.Q.); 2 Department of Chemistry, University of Calgary, 2500 University Dr. NW, Calgary, AB T2N 1N4, Canada; martin.schon@ucalgary.ca (M.A.W.S.); trudels@ucalgary.ca (S.T.)

**Keywords:** additive manufacturing, magnetic composites, ferrite composites, field structuring, microstructure control, rheological modifications

## Abstract

Magnetic composites play a significant role in various electrical and electronic devices. Properties of such magnetic composites depend on the particle microstructural distribution within the polymer matrix. In this study, a methodology to manufacture magnetic composites with isotropic and anisotropic particle distribution was introduced using engineered material formulations and manufacturing methods. An in-house developed material jetting 3D printer with particle alignment capability was utilized to dispense a UV curable resin formulation to the desired computer aided design (CAD) geometry. Formulations engineered using additives enabled controlling the rheological properties and the microstructure at different manufacturing process stages. Incorporating rheological additives rendered the formulation with thixotropic properties suitable for material jetting processes. Particle alignment was accomplished using a magnetic field generated using a pair of permanent magnets. Microstructure control in printed composites was observed to depend on both the developed material formulations and the manufacturing process. The rheological behavior of filler-modified polymers was characterized using rheometry, and the formulation properties were derived using mathematical models. Experimental observations were correlated with the observed mechanical behavior changes in the polymers. It was additionally observed that higher additive content controlled particle aggregation but reduced the degree of particle alignment in polymers. Directionality analysis of optical micrographs was utilized as a tool to quantify the degree of filler orientation in printed composites. Characterization of in-plane and out-of-plane magnetic properties using a superconducting quantum interference device (SQUID) magnetometer exhibited enhanced magnetic characteristics along the direction of field structuring. Results expressed in this fundamental research serve as building blocks to construct magnetic composites through material jetting-based additive manufacturing processes.

## 1. Introduction

Material jetting is an additive manufacturing process where a three-dimensional solid part is manufactured by dispensing polymeric material from a print head and, subsequently, solidifying it utilizing an ultraviolet light or thermal curing methodology. Material jetting employing photopolymerization is similar to stereolithography, where acrylate-type photopolymers are deposited and exposed to ultraviolet light [1]. Promising material systems for future applications involve resins modified with carbon-based fillers, ceramics, and metals [2,3,4,5,6]. Factors that mutually influence the material jetting process are machine and material parameters, including liquid material viscosity, shear-thinning effects and surface tension, print head nozzle design, print speed, and droplet velocity and frequency [7]. The development of fabrication processes includes fine-tuning of material and machine parameters in order to achieve a robust and effective material jetting-based additive manufacturing process [8,9].

**Figure 1:**
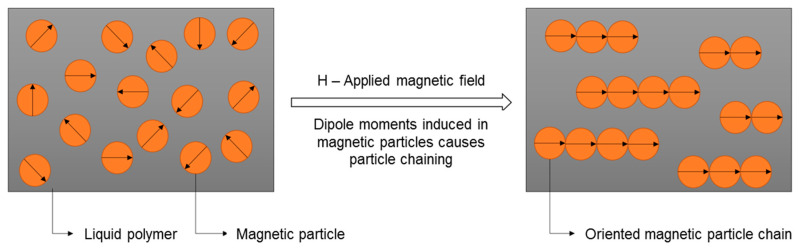
Schematic of magnetic field-induced particle structuring in magnetic polymer composite.

Field-structured magnetic composites are manufactured by aligning magnetic particles within a polymer matrix by applying an external magnetic field. The concept of magnetic field-induced particle structuring is illustrated in Figure 1. Within the polymer matrix, the magnetic particles are randomly distributed after the dispersion process, i.e., random distribution of the crystallographic easy axis of magnetization. When this suspension is subjected to an external magnetic field, magnetic moments are induced along the easy axis, producing particle chaining that enhances magnetic properties like remanence and susceptibility along the direction of field structuring [10,11,12,13]. Such field-structured magnetic materials are of significant interest in applications like magnetic sensors and data storage devices [14,15].

Anisotropy in thermal conductivity has been achieved by orienting ferromagnetic particles in epoxy resin, applying an external magnetic field where the chain-like microstructures serve as enhanced heat flow paths [16]. Inkjet-printed one-dimensional arrays of monodisperse Fe_3_O_4_ nanoparticles with high anisotropic magnetization with possible applications for magnetic field sensing has been demonstrated in the technical literature [17]. Composites 3D printed and poled, applying an external magnetic field, utilizing strontium ferrite particles dispersed in SU8 photoresist, have been observed to be suitable for onboard integration of magnetic components in millimeter-wave circuits [18]. Among the available magnetic materials, ferrite-based magnetic materials have become particularly important for a multitude of applications. Strontium ferrite (SrFe_12_O_19_) is one such material, having a hexagonal structure like magnetoplumbite [19]. Under the influence of an external magnetic field, dipole moments induced in the particles along the crystallographic *c*-axis orient the particles along the direction of the applied magnetic field. Additive manufacturing of magnetorheological fluid-dispersed photopolymers using magnetic field-assisted stereolithography has been studied and reported in the technical literature [20]. The influence of applied magnetic flux density on the degree of particle alignment and orientation behavior at multiple angles has already been reported in previous work by the present authors [21]. The finite element method magnetics (FEMM) simulation for a two-cube magnet particle alignment system has indicated an exponential reduction in magnetic flux density with the increasing separation distance between the magnet faces. Simulations additionally have shown that the magnetic flux density is enhanced by 0.21 Tesla in a magnetic array type particle alignment system compared to a two-cube permanent magnet system [22]. The developed particle alignment systems have been integrated with an in-house developed material jetting 3D printer, which, in addition to material deposition, enables particle structuring during the manufacturing process [23]. Moreover, the effectiveness of utilizing additives to mitigate particle settling in polymers has been reported in the technical literature [24].

The present study investigated the capabilities of polymer formulations engineered with magnetic fillers and additives for the material jetting additive manufacturing process. First, the developed formulations were characterized for their rheological behavior, and mathematical models were employed to derive suspension properties. Derived properties were utilized to correlate different aspects of material and process behavior observed at various manufacturing process stages. The role of additives toward controlling particle aggregation and enabling particle alignment was evaluated using optical microscopy. Optical microscopy, coupled with directionality analysis using image processing, enabled quantifying particle alignment within the dispensed photopolymers. The fundamental understanding thus obtained for developed materials and process scenarios permitted the fabrication of field-structured composites using a suspension engineered with 10 wt% magnetic particle loading. Magnetic characterization of field-structured composites was conducted using a SQUID (superconducting quantum interference device) magnetometer. The goal of this work was to scientifically rationalize material behavior and utilize this knowledge to develop magnetic composite structures with controllable microstructures. Ultimately, this research sought to provide an in-depth understanding of the role of material formulations, magnetic alignment setup, and manufacturing process methods in order to further evolve processes to produce 3D magnetic solids with in-situ microstructure control using the material jetting-based additive manufacturing process.

## 2. Experimental Procedures

### 2.1. Materials

For this study, strontium ferrite (SrFe_12_O_19_, abbreviated as SrFeO) powder with an average particle size of 1.41 μm, density 3.41 g/cm^3^ was purchased from DOWA Electronics Materials Co. Ltd. (Tokyo, Japan). Photosensitive polymer resin PR-48 (UV curable acrylate) was purchased from Colorado Photopolymer Solutions (Boulder, CO, USA), and rheological additive BYK 7410ET was obtained from BYK Additives & Instruments (Wesel, Germany). All chemicals were used as received without any further purification.

### 2.2. Magnetic Filler Dispersion Methodology

The dispersion of magnetic fillers in the UV curable prepolymer was accomplished using a combination of mechanical mixing and sonication. Strontium ferrite powder was added in the desired quantity to the PR-48 resin, and the resultant combination was agitated using an impellor agitator from Calframo Ltd. (Georgian Bluffs, ON, Canada). After mechanical agitation, ultrasonic mixing was initiated using a Branson model S-75 sonicator (Branson Ultrasonics Corporation, Danbury, CT, USA) adopting 15 s p7ulsed on/off mode for 15 min. For experiments involving magnetic particle settling mitigation, the rheological additive was added after the sonication step using mechanical mixing with an impeller. Suspensions were allowed to rest for one day to allow the additive to form a network structure [25]. The suspensions were again mechanically agitated to enhance the efficiency of the rheological additive. Any air that was entrapped during the mixing processes was degassed in a vacuum.

## 3. Characterization Methods

### 3.1. Rheological Behavior—Viscosity and Flow Curve Analysis

Rheological analysis of magnetically loaded prepolymer suspensions containing ferromagnetic particles and rheological additive materials was performed using a rotational rheometer (Rheolab QC, Anton Parr GmbH, Graz, Austria) equipped with double gap type measuring system. Table 1 lists the material formulations that were characterized by their rheological properties.

Flow curves derived from rheological characterization experiments for the magnetic suspensions were used to interpret the suspension behavior. In material jetting processes, extrusion of the developed formulation is driven by applied pressure. Shear forces break the network structure developed in the resin material by the rheological additive. The flow of the liquid formulation that is ejected from the nozzle is primarily governed by equations for incompressible, laminar flow through a circular tube of constant cross-section [26]. The Hagen–Poiseuille equation defines the pressure drop Δp, as indicated in Equation (1) [27].

(1) $$\Delta p=\left(\frac{8\eta QL}{\pi r^{4}}\right)$$

where Q is the volumetric flow rate, η is the formulation viscosity, *r* and *L* are the radius and length of a circular tube, respectively. The shear stress τ at any point inside the circular tube is given by Equation (2) [27].

(2) $$\tau =\;-\frac{\Delta p}{2L}r$$

The wall shear rate, ${\dot{\gamma }}_{w}$, in terms of pressure drop, is given by Equation (3).

(3) $${\dot{\gamma }}_{w}=\;-\frac{\Delta p}{\eta L}\frac{r}{2}$$

The volumetric flow rate *Q* is expressed as [26]:

(4) $$Q=\;\pi r^{2}V=\pi \left(\frac{n}{3n+1}\right){\left(-\frac{\Delta p}{2mL}\right)}^{\frac{1}{n}}\;r^{\frac{3n+1}{n}}$$

In Equation (4), *n* is the power-law index, and *m* is the consistency index, or viscosity, obtained from a mathematical analysis of rheological data through a power-law model. From the theoretical equations, we interpret that $-\Delta p\;\propto \;Q^{n}$, i.e., the pressure gradient is less sensitive for a shear-thinning fluid than for a Newtonian fluid [27]. The dependence of fluid viscosity on the shear rate $\dot{\gamma }$ is expressed utilizing a power law, as indicated in Equation (5) [28].

(5) $$\eta =m{\dot{\gamma }}^{n-1}$$

The magnitude of the power-law index *n* indicates the degree of pseudoplasticity in the characterized material. The power-law model is a two-parameter model and is used extensively to enable a fundamental understanding of fluid behavior. An expression for the actual shear rate experienced by the fluid inside the cylinder, as expressed in the technical literature, is indicated by Equation (6) [26].

(6) $$\dot{\gamma }=\frac{Vr^{\left(2+n\right)}}{\left(\frac{n}{3n+1}\right)r^{\frac{3n+1}{n}}}$$

It has been additionally understood that a formulation with low viscosity experiences higher shear rates during the dispensing process [26]. Overall, from the above theoretical equations, the importance of rheological modification of formulations is well established. The yield strength of the magnetic suspension that characterizes the behavior of the material at rest was determined using the Herschel–Bulkley equation that is well suited for Non-Newtonian fluids. The Hershel–Bulkley model equation is expressed as follows:

(7) $$\tau =\;\tau_{0}+C{\dot{\gamma }}^{k}$$

where *τ*_0_ is the yield point or yield strength, *C* is the consistency index, and *k* is the Herschel–Bulkley index. The Herschel–Bulkley index primarily determines the behavior of the suspension as follows: *k* < 1 for shear thinning behavior, *k* > 1 for shear thickening behavior, and *k* = 1 for Bingham behavior [29,30].

### 3.2. Thixotropic Flow Behavior Analysis

Thixotropy refers to reversible changes in fluid behavior from a flowable liquid to a solid elastic gel. Liquids with a microstructure exhibit thixotropy, given the time needed to reversibly go from a given microstructural state to another. These materials exhibit structural decomposition at high shear rates and structural regeneration at low shear rates. Stresses experienced by the fluid play a dominant role in the breakdown of thixotropic network structures [31]. The influence of rheological additives on the thixotropic behavior of magnetic suspensions was characterized using a step test consisting of three intervals. Low shear rate conditions simulate the sample behavior under stationary conditions, and high shear rate conditions simulate sample behavior under the influence of external forces. Experiments were conducted using controlled shear rate conditions. In the first interval, viscosity was measured at $\dot{\gamma }$ = 1·s^−1^ for 20 s, followed by viscosity measurement at $\dot{\gamma }$ = 300·s^−1^ for 50 s, and finally, viscosity was measured again at $\dot{\gamma }$ = 1·s^−1^ for 40 s. This test was used to characterize the structural decomposition and regeneration behavior of the magnetic suspensions incorporated with rheological additives. The thixotropy index that characterizes time-dependent viscosity recovery was calculated using Equation (8).

(8) $$Thixotropy\;index=\frac{\eta_{2}-\eta_{1}}{t_{2}-t_{1}}=\frac{\Delta \eta }{\Delta t}$$

where $\eta_{2},$ $\eta_{1}$ are viscosities in the recovery phase at two different times $t_{1}$ and $t_{2}$.

### 3.3. Magnetic Particle-Reinforced Resin Behavior in Magnetic Field

To investigate the stability of magnetic particle loaded polymer resin droplets in a magnetic field, an in-house developed experimental system was utilized [21]. A computer control system programmed with a graphical user interface was used to control the mechanical and electrical components of the system. Conditions for the experiments were set using the graphical user interface. The experimental system allowed adjusting the separation between alignment magnets as well as the magnetization time. Droplets were deposited using an Ultimus V deposition system (Nordson EFD, East Providence, RI, USA). Real-time optical microscopy was used to capture the droplet behavior on the substrate.

### 3.4. Magnetic Particle Aggregation Control in Photopolymers

To evaluate the behavior of magnetic particles dispersed in the UV curable polymer, optical microscopy was utilized. The magnetic filler loading in the formulations was maintained at 0.5 wt% to enable light optical microscopy. Particle aggregation due to interparticle magnetic interactions, degree of particle alignment as a function of resin viscosity, and particle chain misorientations were captured and understood in this analysis. Magnetic particles were dispersed in a suspension aggregate due to the magnetic forces that are a function of particle size and magnetization of the particle [31]. Mathematically, the interaction between particles can be characterized using the following equation:

(9) $$W_{\mathrm{mag}}={\;µ}_{0}M^{2}a^{3}$$

where *W*_mag_ is the interaction energy between two magnetic particles, *M* is the particle magnetization, *a* is the particle radius, and µ_0_ is the permeability of a vacuum. To understand the role of additives in controlling particle aggregation, a droplet of the prepared suspension was dispensed within a nylon washer. The state of the particles within the dispensed droplet was captured after 15 min using an optical microscope.

### 3.5. Manufacturing Scenarios for Particle Structuring, and Influence of Resin Viscosity on Particle Alignment

During the process of magnetic particle structuring under an external magnetic field, the dispersed magnetic particles experience forces that are a function of several parameters, i.e., magnetic, gravity, and viscous drag forces. The particle motion is expressed mathematically in Equation (10) [32,33,34,35].

(10) $$\left(\frac{4}{3}\pi a^{3}\rho_{p}\right)\frac{dV_{p}}{dt}=\left[\;F_{m}+F_{d}+F_{g}\;\right]$$

where the individual force terms are expressed as indicated in the following equations:

(11) $$Magnetic\;force:\;F_{m}={\;µ}_{0}\left(\frac{4}{3}\pi a^{3}\right)\chi \frac{1}{2}\;\nabla \left[H\cdot H\right]$$

(12) $$Viscous\;drag\;force:F_{d}=\;6\pi a\eta V_{p}$$

(13) $$Gravitational\;force:\;F_{g}=\left(\frac{4}{3}\pi a^{3}\right)\left(\;\rho_{p}-\rho_{l}\right)g$$

In the above equations, ${µ}_{0}$ is the magnetic permeability of free space, *H* is the magnetic field, *a* is particle radius, *χ* is magnetic susceptibility, *g* is the acceleration due to gravity, *V*_p_ is the particle velocity, *ρ*_p_ and *ρ*_l_ are, respectively, the particle and fluid densities, and *η* is the fluid viscosity. The above equations enable understanding the mechanics of particle alignment, with the most important variables that can be manipulated being the magnetic field (*H*) and resin viscosity (*η*). In previous work, the influence of a magnetic field and novel methods of particle alignment have been already investigated [21]. Simple design changes in the material jetting equipment and alignment methodology have been illustrated, adopting two different manufacturing scenarios. Note that in the original design of the 3D printer, the deposition of the magnetic resin and particle alignment is coupled [23]. Moreover, UV curable resin would cure at the nozzle tip, hindering the deposition process. In the present work, the need for de-coupling printing processes through simple component design changes and manufacturing methodology is described. To understand the influence of resin viscosity on particle alignment, a single layer of the designed sample geometry (15 mm × 15 mm × 1 mm) was dispensed using the material jetting printer and, subsequently, cured using UV light. The height of the magnetic alignment jig from the substrate was maintained at around 10 mm, as further lowering the jig would interfere with the substrate. Optical microscopy was used as a tool to investigate the aforementioned aspects of the experiments. Directionality analysis using ImageJ software (National Institute of Mental Health, Bethesda, MD, USA) was used to quantify the degree of particle alignment in field-structured composites [36].

### 3.6. Additive Manufacturing of Magnetic Polymer Composites and Magnetic Characterization

To manufacture field-structured magnetic composites, a material jetting 3D printer was utilized to deposit the ferromagnetic resin on the substrate. An in-house developed material jetting 3D printer controlled using the LabVIEW programming environment (National Instruments, Austin, TX, USA) was employed to deposit the material to the designed geometry. A graphical user interface enabled controlling the different movements of the 3D printer. The sample geometry was designed in SolidWorks (Dassault Systems, Vélizy-Villacoublay, France), and the open-source software Sli3cr was used to generate the G-code for the nozzle deposition path [37]. The generated G-code was further modified for proper positioning of the magnetic alignment jig above the deposited material and, subsequently, curing every deposited layer.

The field-structured magnetic composite magnetic properties were characterized using a SQUID magnetometer (MPMS XL-7 Evercool, Quantum Design, San Diego, CA, USA) for its in-plane and out-of-plane magnetic characteristics, i.e., magnetic properties were measured along and perpendicular to the direction of particle structuring. A small piece from a 3D printed part was first weighed, placed in a gelatin capsule, and further inserted into a transparent diamagnetic plastic straw. Magnetization reversal loops were performed by applying a magnetic field with a strength of µ_0_H = ± 7 Tesla at a temperature of 300 K. Saturation magnetization was determined at an applied field strength of 7 Tesla. Remanence and coercivity were obtained, respectively, at zero applied field and as the applied field yielding zero magnetization from the hysteresis data. The magnetic properties were determined by averaging the values obtained through both magnetization and de-magnetization cycles.

## 4. Results and Discussion

### 4.1. Rheological Behavior Analysis of Ferromagnetic Polymers

**Figure 2:**
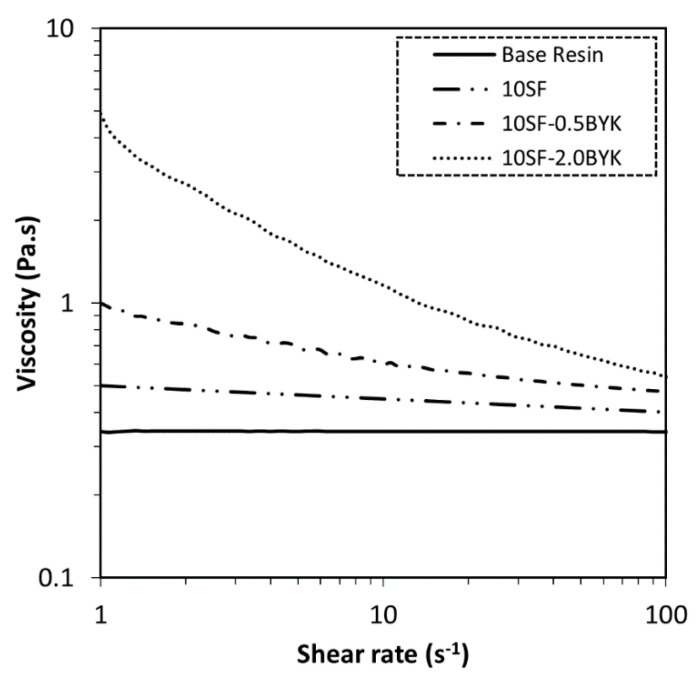
Viscosity as a function of shear rate for material formulation listed in Table 1.

Rheological properties of filler-modified resin formulations have great significance in extrusion-based additive manufacturing processes [38,39]. In the present rheological study, changes in material behavior as a result of two different additive loadings are investigated. Materials developed and utilized for additive manufacturing processes are subjected to various types of shear rates and deformations during storage and/or the manufacturing process. The study of viscosity properties at different shear rates provides useful information on the properties of the developed formulations. It is well-known that the rheological properties of the polymer formulations strongly depend on the characteristics of the fillers, volume fraction of fillers, dispersion quality, and network structure within the polymer [40,41]. As observed in Figure 2, apart from the base resin (pure PR-48) that exhibits Newtonian behavior (viscosity is independent of shear rate), all other formulations exhibit non-Newtonian behavior, which is confirmed by observing a decrease in viscosity with increasing shear rates. First, adding strontium ferrite to the base resin increases the viscosity of the suspension and makes the mixture exhibit non-Newtonian behavior. Additionally, an increase in low shear viscosity is observed in suspensions modified using the rheological additive. It is observed that the additive loading significantly influences the magnitude of an increase in low shear viscosity. Such observations where changes in mechanical behavior are imposed on the resin system by introducing additives are deemed important for extrusion-based additive manufacturing processes [42]. The BYK 7410ET additive is a polyuria-based thixotropic additive material system. Such material, when dispersed in a polymeric matrix, results in the formation of a network structure by hydrogen bonding [43]. The development of a structural network within the polymeric binder is supported by the enhancement in low shear (at 1·s^−1^) viscosity observed in Figure 2. With increasing shear rate, the viscosity decreases due to the disruption of this structural network. By fitting the viscosity versus shear rate data to a power-law model (using Origin software, Version 2020, OriginLab Corporation, Northampton, MA, USA), the formulation properties (power-law index and viscosity) are determined to understand the influence of additive loading (see Table 2).

As observed in Table 2, curve fitting using the power-law model for all the formulations except for the base resin confirms shear thinning or pseudoplastic material behavior with power-law index *n* < 1. Additionally, it is observed that the additive loading significantly influences the degree of pseudoplasticity. The 10SF-2.0BYK formulation with the lowest power-law index exhibits the highest material viscosity, which is advantageous for extrusion-based additive manufacturing.

Yield point or yield stress, defined as the shear stress at zero shear rate, determined by curve fitting of shear stress versus shear rate data using Equation (7) are listed in Table 3. With Herschel–Bulkley indices being less than unity, it is additionally confirmed that all suspensions exhibit pseudoplastic behavior. As far as the yield strengths of the suspensions are concerned, they are observed to be dependent on additive loading. The additive is found to be efficient in terms of yield strength enhancement, as indicated by the results shown in Table 3. The enhancement in yield strength is caused by the thixotropic network structure as a result of additive incorporation within the magnetic particle reinforced formulations.

### 4.2. Thixotropic Flow Behavior Analysis

**Figure 3:**
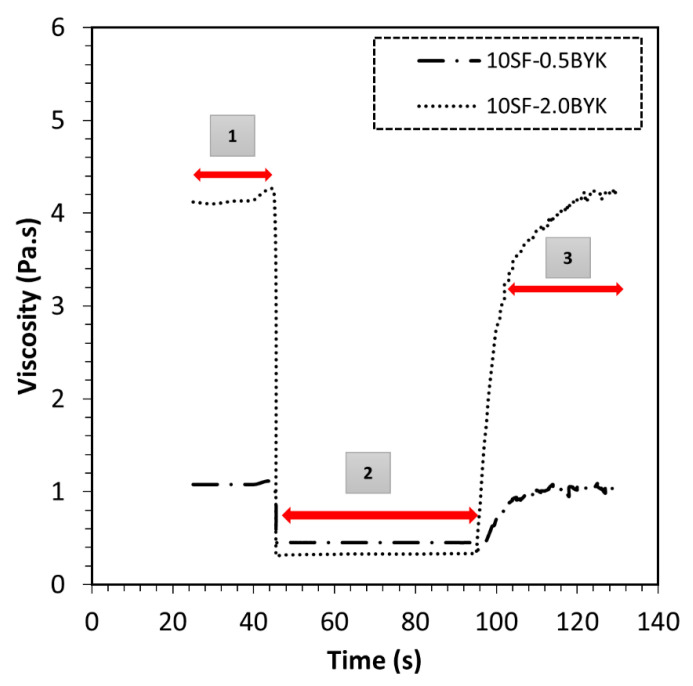
Three interval thixotropy tests for developed material formulations (1: Low shear phase $\dot{\gamma }$ = 1·s^−1^; 2: High shear phase $\dot{\gamma }$ = 300·s^−1^; 3: Low shear phase $\dot{\gamma }$ = 1·s^−1^).

In material jetting additive manufacturing processes, the material experiences various forces at different processing stages, i.e., forces are imposed on the material during handling, pressure-induced material dispensing, and magnetic field exposure during particle structuring. Thixotropy analysis is further utilized to understand and determine the viscosity recovery in the magnetic suspensions. Results of the step test consisting of three intervals shown in Figure 3 indicate high initial suspension viscosity at $\dot{\gamma }\;$= 1·s^−1^, followed by instantaneous viscosity reduction when the shear rate is increased to $\dot{\gamma }$ = 300·s^−1^. Finally, in the recovery phase, when the shear rate is again reduced to $\dot{\gamma }$ = 1 s^−1^, the magnetic suspensions exhibit time-dependent viscosity recovery behavior. The observed phenomena relate to the processes of structural decomposition at high shear rates and structural regeneration at low shear rates that are mainly controlled by the rheological additives. From this analysis, it is well understood that the additive imparts thixotropic properties to the magnetic suspension. Thixotropy indices calculated using Equation (8) are listed in Table 4.

The step test data indicates a strong influence of rheological additive content on the magnitude of viscosity recovery. This type of structural decomposition and regeneration is deemed to be one of the fundamental requirements for a material to be considered for material jetting-based additive manufacturing. Present results corroborate findings in the technical literature [26].

### 4.3. Magnetic Particle-Reinforced Resin Behavior in Magnetic Field

**Figure 4:**
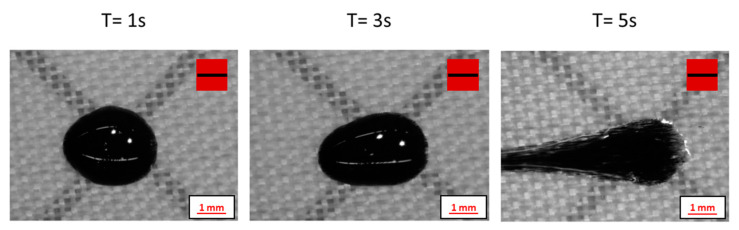
Optical microscopy images of droplet deformation for different magnetization times at magnet separation distance of 30 mm (Formulation: 10SF). Red squares with a black bar indicate the direction of the magnetic field.

In this study, the behavior of magnetically loaded polymer droplets is evaluated as a function of time at a magnet separation distance of 30 mm, where the magnetic flux density at the center between two magnets is 0.10 Tesla. From particle alignment experiments, it is evident that the degree of particle alignment is higher at a separation distance of 30 mm between the cube magnets [10]. However, it is observed that droplets dispensed on the substrate deform significantly under the influence of the magnetic field. Experiments are conducted using the base resin reinforced with just 10 wt% SrFeO particles to determine the time at which a droplet loses its stability on the substrate. Deformation of magnetic particle-reinforced polymer droplets obtained from experiments conducted, varying the time at a separation distance of 30 mm, is depicted in Figure 4.

**Figure 5:**
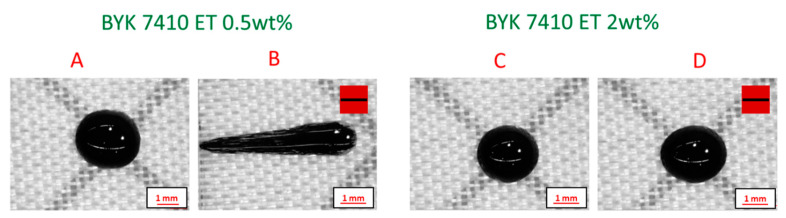
Droplet images, showing the influence of rheological additive loading: (**A**) and (**C**): Droplet images before magnetic field application; (**B**) and (**D**): Droplet images after field application. Red squares with a black bar indicate the presence and direction of the magnetic field.

The magnetic particle-reinforced resins engineered with 10 wt% magnetic particles loading and 0.5 wt% and 2 wt% of BYK 7410ET are tested for their stability on the substrate. As observed in Figure 5, the resin engineered with higher additive loading (2 wt%) is stable on the substrate in the presence of a magnetic field. This observed behavior corresponds well with the rheological analysis results, where the formulation engineered using 2 wt% of the additive exhibits enhanced low shear viscosity, yield strength, and thixotropic properties.

### 4.4. Magnetic Particle Aggregation Control in Photopolymers

The present study also investigates the ability of magnetic particle-reinforced formulations to control particle aggregation due to interparticle interactions. To enable optical microscopy, the magnetic filler loading is kept low at 0.5 wt%. Table 5 lists the formulations prepared for evaluating the magnetic particle behavior in the prepared formulations.

**Figure 6:**
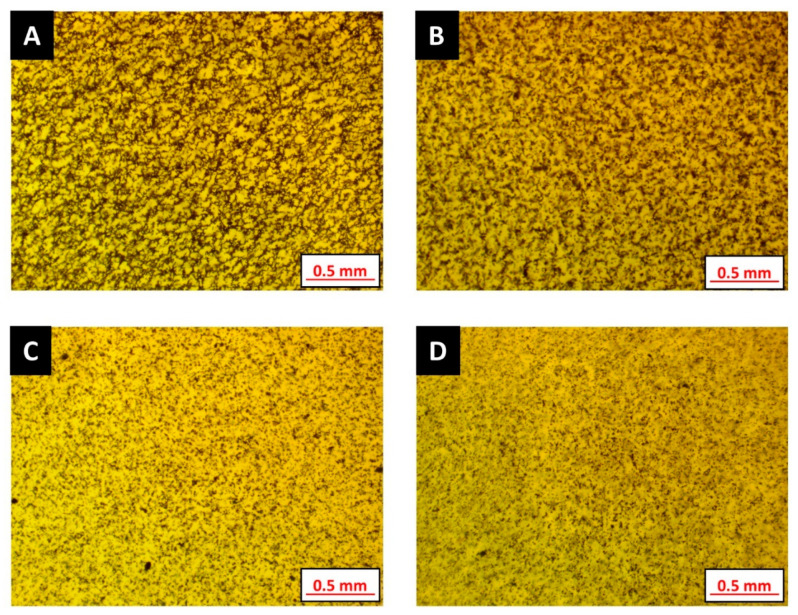
Particle aggregation decreasing from (**A**–**D**) in photosensitive polymer formulations, as listed in Table 5, (**A**), (**B**), (**C**) and (**D**) have different BYK 7410ET additive loading separately.

First, the influence of additive loading toward mitigating particle aggregation is taken into consideration. According to Equation (9), magnetic particles attract each other due to the interaction energy and tend to form aggregates. The formation of aggregates is evident in the micrographs shown in Figure 6A,B. Aggregation is observed to reduce with increasing additive content in the base resin. Even though the micrograph in Figure 6B shows particle aggregation, the randomized chaining of magnetic particles is not as profound as the formulation without any additive (Figure 6A). Micrographs in Figure 6C,D exhibit uniform particle dispersion as the viscous drag due to the additive inhibits magnetic particle motion. This viscous drag is a result of thixotropic network formation within the formulation. The plastic fluidity, which is a result of additive incorporation, ensures good dispersion of the magnetic particles within the developed photopolymer formulation.

### 4.5. Manufacturing Scenarios and Influence of Resin Viscosity on Particle Alignment

**Figure 7:**
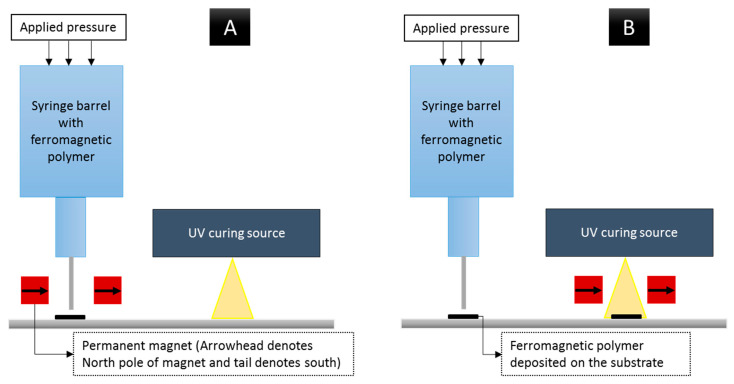
Graphical representation of adopted manufacturing scenarios: (**A**) field structuring setup decoupled from curing process, and (**B**) the alignment system coupled with curing source.

**Figure 8:**
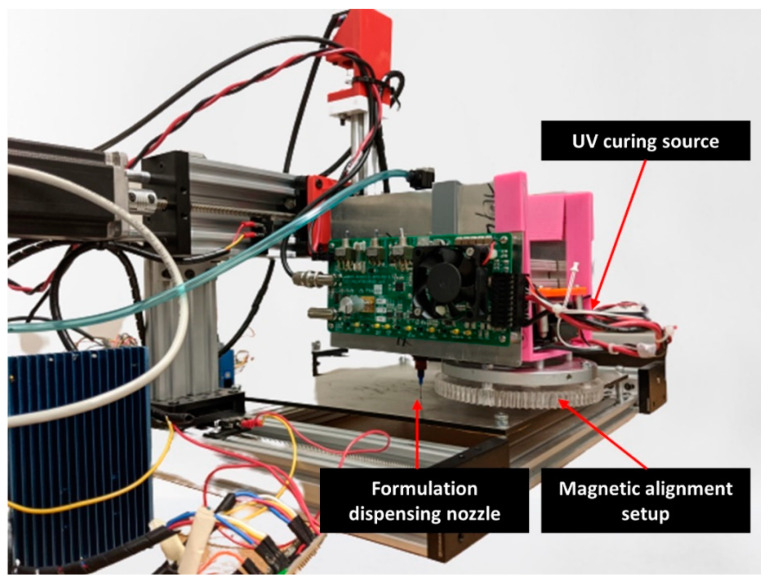
Material jetting equipment with the in-situ particle alignment system, representing manufacturing scenario (B) in Figure 7.

**Figure 9:**
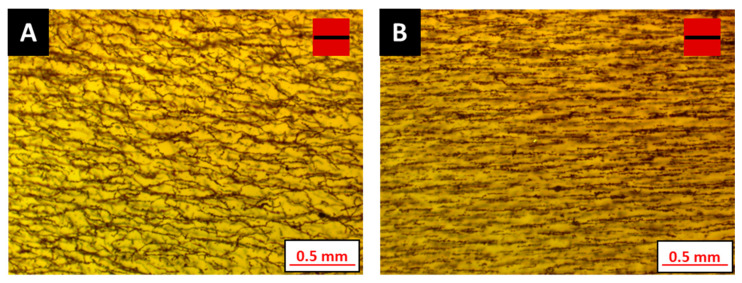
Micrographs obtained from the experiments adopting machine configuration scenarios (**A**) and (**B**), as indicated in Figure 7. Red squares with a black bar indicate the direction of the magnetic field.

To manufacture field-structured magnetic composites, two different manufacturing scenarios are tested. In scenario A, represented in Figure 7A, the permanent magnet alignment system is coupled with the dispensing system. In this manufacturing scenario, when the dispensing nozzle is in the presence of the magnetic field, some manufacturing issues, i.e., nozzle clogging, are encountered. These fabrication issues are overcome by adjusting the dispensing pressure. Once dispensed, the curing source moves to the dispensed material and solidifies the resin. In scenario B, depicted in Figure 7B, the alignment system is coupled with the curing system. Figure 8 shows the manufacturing system configuration of scenario B for the developed material jetting system. All machine movements are accomplished by programming appropriate G-codes with the respective wait times prior to the curing process. From the micrograph shown in Figure 9, it is understood that the alignment system coupled with the curing source enhances particle structuring (Figure 9B), whereas removing the field structuring setup during the curing process (Figure 9A) results in chain misalignment and a reconfigured microstructure.

**Figure 10:**
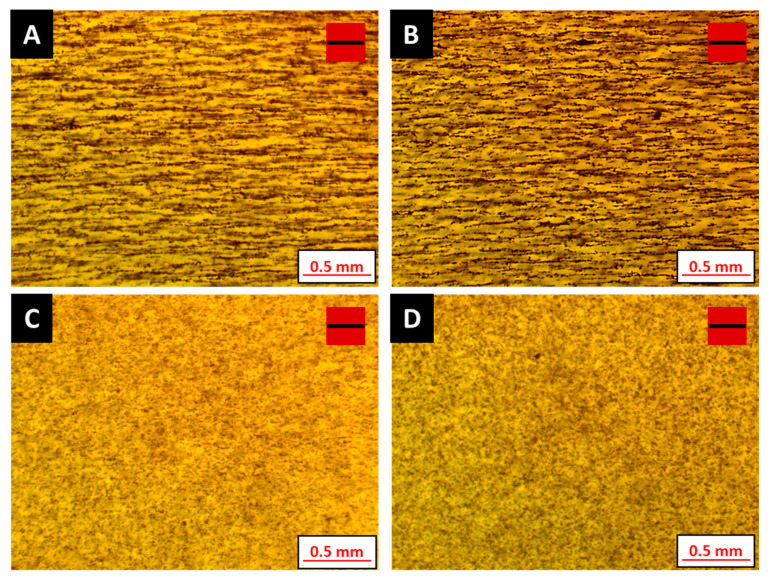
Influence of additive loading on particle alignment (**A**–**D** refer to suspensions listed in Table 5). Red squares with a black bar indicate the presence and direction of the magnetic field.

**Figure 11:**
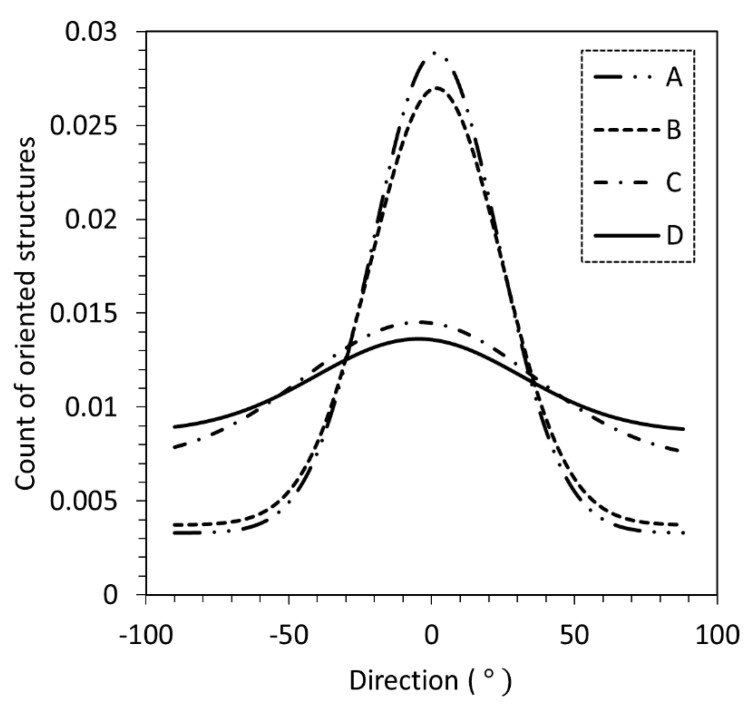
Particle alignment orientation obtained using image analysis (**A**–**D** refer to suspensions listed in Table 5).

The influence of additives on the degree of particle alignment is also studied, adopting scenario B as the machine setting and dispensing one single layer of the designed sample geometry. The height of the magnetic alignment jig is maintained at a target of 10 mm from the substrate. Maintaining this height requires increasing the time to allow particle chaining to occur prior to UV curing. The wait time prior to curing is maintained at 60 s. Optical micrographs obtained for samples manufactured using the formulations listed in Table 5 are shown in Figure 10. Results from the directionality analysis shown in Figure 11 indicate that increasing the additive loading decreases the particle chaining effect. The count of oriented structures, which enables quantification of filler directionality and the degree of filler orientation, is observed to decrease with increasing additive content. Furthermore, the observed behavior emphasizes that magnetic field strength, along with resin viscosity, are critical aspects of the manufacturing process, especially concerning the degree of particle alignment.

### 4.6. Additive Manufacturing of Field-Structured Composites and Magnetic Characterization

**Figure 12:**
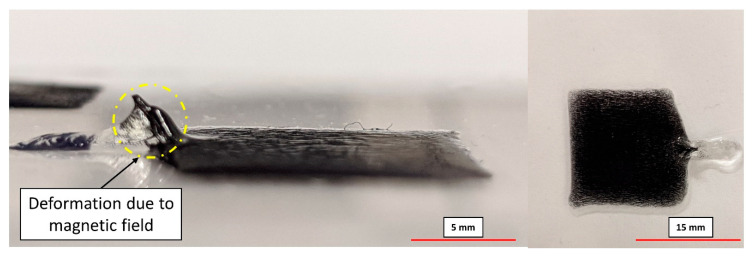
3D printed magnetic composite with observed deformations caused by the magnetic field (deformation indicated by circles).

Magnetic field-structured composites are printed using a formulation engineered with 10 wt% magnetic filler and 0.5 wt% BYK 7410ET additive. Printing magnetic composites is restricted to a simple geometry (flat plate, as indicated in Figure 12), as the primary focus of this study is to develop composites with field-structured microstructure. Additionally, experiments conducted at lower filler loadings enable establishing apt rheological additive loading and manufacturing scenarios as validated through optical microscopy. The extrusion pressure is set at 17 kPa (2.5 psi); layer thickness and deposition speed are 0.2 mm and 10 mm/s, respectively. Printer settings are modified to position the magnetic alignment jig and the curing source right above the deposited material. Such modifications are done to fabricate composite samples with three print layers of the deposited material. The times for magnetic field application and UV irradiation are maintained at 60 s and 20 s for every layer, respectively. As shown in Figure 12, small deformations caused by the magnetic field during the alignment process prior to curing are observed. These deformations occur due to a non-uniform magnetic flux density within the magnetic jig, which has been already reported in previous work [21].

**Figure 13:**
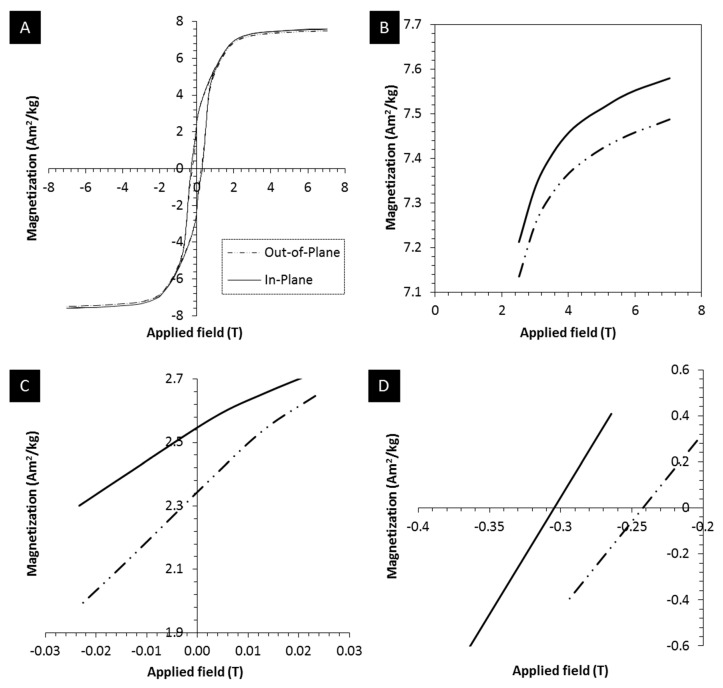
(**A**): Hysteresis data for magnetization versus applied magnetic field; (**B**), (**C**), and (**D**): Magnified views of hysteresis graph for saturation magnetization, magnetic remanence, coercivity, respectively.

Magnetic composites are characterized by properties like saturation magnetization, magnetic remanence, and coercivity. These magnetic characteristics are derived from magnetization reversal loops. Figure 13A shows the magnetization reversal data for a sample evaluated along (in-plane) and perpendicular (out-of-plane) to the direction of particle structuring.

The first observed characteristic in Figure 13A is the presence of hysteresis, attributed to the ferromagnetic SrFeO filler. The saturation magnetization of ~7.5 Am^2^/kg is consistent with the 10% of SrFeO with a saturation magnetization of ~70 Am^2^/kg, as previously reported [30]. Compared to the direction perpendicular to particle structuring, saturation magnetization along the direction of field structuring is observed to be higher by 0.10 Am^2^/kg, magnetic remanence by 0.13 Am^2^/kg, and coercivity by 0.06 Tesla, see Figure 13B–D, respectively. This behavior of remanence and coercivity is consistent with a magnetic easy axis along the direction of particle structuring and a magnetic hard axis perpendicular to it. This anisotropy is likely a combined effect of magnetocrystalline easy axis orientation, as well as shape anisotropy due to the chain-like configuration of the magnetic particles in the cured resin. The difference in saturation magnetization is most likely due to the samples not reaching full saturation even at 7 Tesla applied field, which is conceivable for magnetically highly anisotropic SrFeO particles. Nonetheless, the data indicates that magnetization is easier to achieve along the structuring direction. The area enclosed by the hysteresis curve for measurements along the direction of particle structuring is greater by approximately 5% compared to the hysteresis curve obtained for the direction perpendicular to the direction of field structuring. The present observations and findings are congruent with results in the technical literature for magnetic composites with aligned magnetic fillers [33,44]. This enhancement further confirms the alignment of the easy axis of magnetization of individual particles by orienting the fillers, resulting in the chain-like microstructures. Overall, magnetic composite structures printed by orienting ferromagnetic particles exhibit anisotropic magnetic properties due to particle assembly through the external magnetic field, which has the potential to unlock innovative approaches to build magnetic structures for a wide range of applications.

## 5. Conclusions

The present study expanded the understanding of material formulations for magnetic polymer composites in a multidisciplinary context. Rheology studies enabling microstructure control, field-structured composite manufacturing scenarios, and magnetic characterization of field-structured composites were successfully performed. It was observed that rheological additive materials enabled enhancing the low shear viscosity and yield strength through the formation of a thixotropic network within the prepared formulations. Mathematical analysis of rheological data enabled interpreting the formulation properties like flow index and yield strength. A flow index of less than unity for all formulations provided strong evidence for pseudoplastic material behavior. Additionally, a step test consisting of three intervals demonstrated the time-dependent viscosity recovery in the magnetic suspensions. It was observed that fluid properties like flow index, yield strength, and thixotropy index were dependent on the additive loading in the developed formulations. Experiments conducted using a formulation engineered with BYK 7410ET additive revealed that at the highest additive loading of 2 wt%, the deposited resin material was stable on the substrate in the presence of the magnetic field. However, optical microscopy at lower magnetic filler loading revealed that an increase in additive loading, while suppressing aggregation of magnetic particles, severely reduced the desired chaining effect in the presence of an applied magnetic field due to enhanced viscous drag in the magnetic formulations. Optical microscopy, coupled with image processing, enabled quantifying the degree of particle orientation in the polymer formulations. These fundamental studies were critical for providing an understanding of the role of material formulations in achieving a variety of material and manufacturing process-related goals for the additive manufacturing process in order to create magnetic particle-reinforced composites. Composites characterized using SQUID magnetometry revealed an enhancement in magnetic properties along the direction of particle structuring. Compared to the out-of-plane magnetic characteristics, the in-plane magnetic saturation, remanence, and coercive fields were observed to be enhanced. Ultimately, this research work provides the basis for devising robust material formulations and manufacturing processes to effectively form magnetic polymer composites with desired microstructure distribution during material jetting-based additive manufacturing.

## Figures and Tables

**Table 1:** Materials characterized by rheological properties.

Material Code	Magnetic Filler Loading (wt%)	Additive Type	Additive Loading (wt%)
Base resin	-	-	-
10SF	10	-	-
10SF-0.5BYK	10	BYK-7410ET	0.5
10SF-2.0BYK	10	BYK-7410ET	2

**Table 2:** Rheological properties derived using Equation (5).

Material Code	*n* - Power Law Index	*m* - Viscosity (Pa·s)
Base resin	-	-
10SF	0.95	0.50
10SF-0.5BYK	0.87	0.88
10SF-2.0BYK	0.61	3.23

**Table 3:** Yield stress predictions using the Herschel–Bulkley model (Equation (7)).

Material Code	Herschel–Bulkley Model predictions		
	Yield Strength - $\tau_{0}$ (Pa)	Consistency index - C (Pa·s)	Herschel-Bulkley index - k
Base resin	-	-	-
10SF	0.26	0.45	0.97
10SF-0.5BYK	0.65	0.59	0.93
10SF-2.0BYK	3.07	1.18	0.81

**Table 4:** Thixotropy index of developed magnetic suspensions.

Material Code	Thixotropy Index
10SF-0.5BYK	0.05
10SF-2.0BYK	0.32

**Table 5:** Suspensions prepared for optical microscopy analysis.

Sample Identifier	Magnetic Filler Loading (wt%)	BYK 7410ET Additive Loading (wt%)
A	0.5	0
B	0.5	0.5
C	0.5	1.0
D	0.5	2.0
